# Graphical abstracts in *British Journal of Nutrition* – ADDENDUM

**DOI:** 10.1017/S0007114523001095

**Published:** 2023-12-14

In the below articles published in the *British Journal of Nutrition*, the graphical abstract was erroneously omitted at the time of publication. The corresponding graphical abstract has now been added to the following articles and is present in their HTML version.

Cambridge University Press apologises for this error.

## References

[ref1] Pagliai, G. , Dinu, M. , Madarena, M. , Bonaccio, M. , Iacoviello, L. , & Sofi, F. (2021). Consumption of ultra-processed foods and health status: A systematic review and meta-analysis. British Journal of Nutrition, 125(3), 308–318. doi: 10.1017/S0007114520002688 32792031 PMC7844609

[ref2] Bermano, G. , Méplan, C. , Mercer, D. , & Hesketh, J. (2021). Selenium and viral infection: Are there lessons for COVID-19? British Journal of Nutrition, 125(6), 618–627. doi: 10.1017/S0007114520003128 32758306 PMC7503044

[ref3] Richardson, D. , & Lovegrove, J. (2021). Nutritional status of micronutrients as a possible and modifiable risk factor for COVID-19: A UK perspective. British Journal of Nutrition, 125(6), 678–684. doi: 10.1017/S000711452000330X 32815493 PMC7492581

[ref4] Atkins, J. , & Wannamathee, S. (2020). Sarcopenic obesity in ageing: Cardiovascular outcomes and mortality. British Journal of Nutrition, 124(10), 1102–1113. doi: 10.1017/S0007114520002172 32616084

[ref5] Gete, D. , Waller, M. , & Mishra, G. (2020). Effects of maternal diets on preterm birth and low birth weight: A systematic review. British Journal of Nutrition, 123(4), 446–461. doi: 10.1017/S0007114519002897 31711550

[ref6] Virgens, I. , Santana, N. , Lima, S. , & Fayh, A. (2021). Can COVID-19 be a risk for cachexia for patients during intensive care? Narrative review and nutritional recommendations. British Journal of Nutrition, 126(4), 552–560. doi: 10.1017/S0007114520004420 33261670 PMC7711335

[ref7] Pranata, R. , Feraldho, A. , Lim, M. , Henrina, J. , Vania, R. , Golden, N. , & July, J. (2022). Coffee and tea consumption and the risk of glioma: A systematic review and dose–response meta-analysis. British Journal of Nutrition, 127(1), 78–86. doi: 10.1017/S0007114521000830 33750490

[ref8] Neale, E. , Guan, V. , Tapsell, L. , & Probst, Y. (2020). Effect of walnut consumption on markers of blood glucose control: A systematic review and meta-analysis. British Journal of Nutrition, 124(7), 641–653. doi: 10.1017/S0007114520001415 32312354

[ref9] Fang, C. , Feng, L. , Jiang, W. , Wu, P. , Liu, Y. , Kuang, S. , … Zhou, X. (2021). Effects of dietary methionine on growth performance, muscle nutritive deposition, muscle fibre growth and type I collagen synthesis of on-growing grass carp (Ctenopharyngodon idella). British Journal of Nutrition, 126(3), 321–336. doi: 10.1017/S0007114520002998 32718370

[ref10] Hutchins-Wiese, H. , Bales, C. , & Porter Starr, K. (2022). Mediterranean diet scoring systems: Understanding the evolution and applications for Mediterranean and non-Mediterranean countries. British Journal of Nutrition, 128(7), 1371–1392. doi: 10.1017/S0007114521002476 34289917

[ref11] Marreiro, D. , Cruz, K. , Oliveira, A. , Morais, J. , Freitas, B. , Melo, S. , … Dias, T. (2022). Antiviral and immunological activity of zinc and possible role in COVID-19. British Journal of Nutrition, 127(8), 1172–1179. doi: 10.1017/S0007114521002099 34128459 PMC8438509

[ref12] Daniel, H. (2020). Diet and the gut microbiome: From hype to hypothesis. British Journal of Nutrition, 124(6), 521–530. doi: 10.1017/S0007114520001142 32238220

[ref13] Wierzbicka, A. , & Oczkowicz, M. (2022). Sex differences in vitamin D metabolism, serum levels and action. British Journal of Nutrition, 128(11), 2115–2130. doi: 10.1017/S0007114522000149 35042577

[ref14] Fillon, A. , Beaulieu, K. , Mathieu, M. , Tremblay, A. , Boirie, Y. , Drapeau, V. , & Thivel, D. (2021). A systematic review of the use of the Satiety Quotient. British Journal of Nutrition, 125(2), 212–239. doi: 10.1017/S0007114520002457 32616106

[ref15] Liang, Y. , Zou, L. , Tian, Y. , Zhou, S. , Chen, X. , & Lin, C. (2021). Dietary and metabolic risk of neuropsychiatric disorders: Insights from animal models. British Journal of Nutrition, 126(12), 1771–1787. doi: 10.1017/S0007114521000659 33618780

[ref16] Zhang, Z. , Luo, B. , Xu, S. , Zhang, Z. , Xing, W. , Chen, Y. , … Xu, D. (2021). Long-term vitamin D deficiency promotes renal fibrosis and functional impairment in middle-aged male mice. British Journal of Nutrition, 125(8), 841–850. doi: 10.1017/S0007114520003232 32812524

[ref17] Rosignoli da Conceição, A. , Dias, K. , Michelin Santana Pereira, S. , Saraiva, L. , Alves Oliveira, L. , Gomes de Souza, E. , … Mattos Della Lucia, C. (2022). Protective effects of whey protein concentrate admixtured of curcumin on metabolic control, inflammation and oxidative stress in Wistar rats submitted to exhaustive exercise. British Journal of Nutrition, 127(4), 526–539. doi: 10.1017/S0007114521001355 33902765

[ref18] Bailly, M. , Germain, N. , Galusca, B. , Courteix, D. , Thivel, D. , & Verney, J. (2020). Definition and diagnosis of constitutional thinness: A systematic review. British Journal of Nutrition, 124(6), 531–547. doi: 10.1017/S0007114520001440 32321597

[ref19] Clark, J. , Simpson, B. , & Murphy, K. (2022). The role of a Mediterranean diet and physical activity in decreasing age-related inflammation through modulation of the gut microbiota composition. British Journal of Nutrition, 128(7), 1299–1314. doi: 10.1017/S0007114521003251 34423757

[ref20] Akbarzade, Z. , Djafarian, K. , Saeidifard, N. , Aliakbari Majd, S. , Garousi, N. , Samadi, F. , … Shab-Bidar, S. (2022). The association between lunch composition and obesity in Iranian adults. British Journal of Nutrition, 127(10), 1517–1527. doi: 10.1017/S0007114521002543 34236018

[ref21] Yeh, W. , Ko, J. , Cheng, W. , & Yang, H. (2022). Diet containing dehulled adlay ameliorates hepatic steatosis, inflammation and insulin resistance in rats with non-alcoholic fatty liver disease. British Journal of Nutrition, 128(3), 369–376. doi: 10.1017/S0007114521003366 34470675

[ref22] Shrestha, N. , Sleep, S. , Helman, T. , Holland, O. , Cuffe, J. , Perkins, A. , … Hryciw, D. (2022). Maternal diet high in linoleic acid alters offspring fatty acids and cardiovascular function in a rat model. British Journal of Nutrition, 127(4), 540–553. doi: 10.1017/S0007114521001276 33858529

[ref23] Nørgaard, S. , Dalgård, C. , Heidemann, M. , Schou, A. , & Christesen, H. (2021). Bone mineral density at age 7 years does not associate with adherence to vitamin D supplementation guidelines in infancy or vitamin D status in pregnancy and childhood: An Odense Child Cohort study. British Journal of Nutrition, 126(10), 1466–1477. doi: 10.1017/S0007114521000301 33494857 PMC8524427

[ref24] Lin, Y. , Chen, M. , Peng, Y. , Chen, Q. , Li, S. , & Chen, L. (2021). Feeding intolerance and risk of poor outcome in patients undergoing cardiopulmonary bypass surgery. British Journal of Nutrition, 126(9), 1340–1346. doi: 10.1017/S0007114521000167 33468265 PMC8505710

[ref25] Griffiths, A. , Matu, J. , Whyte, E. , Akin-Nibosun, P. , Clifford, T. , Stevenson, E. , & Shannon, O. (2022). The Mediterranean dietary pattern for optimising health and performance in competitive athletes: A narrative review. British Journal of Nutrition, 128(7), 1285–1298. doi: 10.1017/S0007114521003202 34420536

[ref26] Daniel, H. , Hauner, H. , Hornef, M. , & Clavel, T. (2022). Allulose in human diet: The knowns and the unknowns. British Journal of Nutrition, 128(2), 172–178. doi: 10.1017/S0007114521003172 34409930

[ref27] Michalikova, D. , Tyukos Kaprinay, B. , Brnoliakova, Z. , Sasvariova, M. , Krenek, P. , Babiak, E. , … Gasparova, Z. (2021). Impact of improving eating habits and rosmarinic acid supplementation on rat vascular and neuronal system in the metabolic syndrome model. British Journal of Nutrition, 125(7), 757–767. doi: 10.1017/S000711452000327X 32814604

[ref28] Szwiega, S. , Pencharz, P. , Ball, R. , Tomlinson, C. , Elango, R. , & Courtney-Martin, G. (2022). Amino acid oxidation methods to determine amino acid requirements: Do we require lengthy adaptation periods? British Journal of Nutrition, 1-7. doi: 10.1017/S0007114522002720 PMC10167660 36045125

[ref29] Kiriyama, K. , Yamamoto, M. , Kim, D. , Sun, S. , Yamamoto, H. , & Oda, H. (2022). Skipping breakfast regimen induces an increase in body weight and a decrease in muscle weight with a shifted circadian rhythm in peripheral tissues of mice. British Journal of Nutrition, 128(12), 2308–2319. doi: 10.1017/S0007114522000356 35272720

[ref30] Oliva, L. , Alemany, M. , Fernández-López, J. , & Remesar, X. (2022). Circulating oestradiol determines liver lipid deposition in rats fed standard diets partially unbalanced with higher lipid or protein proportions. British Journal of Nutrition, 128(8), 1499–1508. doi: 10.1017/S0007114521004505 34776031 PMC9557166

[ref31] Hajizadeh-Sharafabad, F. , Sharifi Zahabi, E. , & Tarighat-Esfanjani, A. (2022). Role of whey protein in vascular function: A systematic review and meta-analysis of human intervention studies. British Journal of Nutrition, 128(4), 659–672. doi: 10.1017/S0007114521003676 34511143

